# The Utility of Intraoperative Near Infrared Fluorescence (NIR) Imaging with Indocyanine Green (ICG) for the Assessment of Kidney Allograft Perfusion

**DOI:** 10.1155/2018/6703056

**Published:** 2018-08-19

**Authors:** Edwin Jonathan Aslim, Fang Jann Lee, Valerie Huei Li Gan

**Affiliations:** ^1^Department of Urology, Singapore General Hospital, Singapore; ^2^Singapore Medical Specialists Centre, Singapore

## Abstract

**Background:**

Near infrared light (NIR) fluorescence imaging with indocyanine green (ICG) has been used in various aspects of surgery, such as in the assessment of vascular anastomosis, tissue perfusion, and the identification of lymph nodes. In this study we evaluated the utility of NIR/ICG fluorescence imaging in kidney transplantation.

**Materials and Methods:**

NIR/ICG imaging was used to assess allograft perfusion in n=1 living donor (LDRT) and n=2 deceased donor (DDRT) renal transplantations, performed in February 2017. The allograft arterial and venous anastomoses were done end-to-side to the corresponding recipient external iliacs, and ureteroneocystostomies were performed for urinary reconstructions. After completion of vascular anastomosis, ICG was given as intravenous bolus at 0.3mg/kg, followed by visual assessment of tissue perfusion and vascular anastomoses at 1-minute interval using fluorescence imaging (KARL STORZ NIR/ICG System).

**Results:**

Homogenous global fluorescence of the allograft and vascular anastomosis was observed in all 3 cases. Immediate postoperative perfusion studies showed patent inflow and outflow vessels and well perfused transplanted kidneys. Immediate graft function was observed in 2 recipients (1 LDRT and 1 DDRT). One session of haemodialysis was performed in 1 DDRT recipient, for high serum potassium in the immediate postoperative setting, who otherwise had good urine output and serially declining serum creatinine.

**Conclusions:**

NIR/ICG fluorescence imaging can be useful in renal transplantation for the intraoperative assessment of allograft perfusion, especially in complex cases with multiple renal arteries and vascular reconstructions.

## 1. Introduction

Advances in near infrared (NIR) imaging technology have expanded the use of fluorescence imaging in live surgery, with indocyanine green (ICG) being the most widely used fluorophore. NIR/ICG imaging has been used in various aspects of oncological and reconstructive operations, including identification of tumours for resections, assessment of vascular anastomosis and tissue perfusion, and lymph node dissections [1–5]. More recently, NIR/ICG imaging has also been used in laparoscopic and robotic surgeries [5–7]. There are limited reports in the literature on the role of fluorescence imaging in kidney transplantation [8–10]. While intraoperative Doppler ultrasound can give adequate information on the status of vascular anastomosis, demonstrating satisfactory global allograft perfusion can be tedious, especially in cases with challenging vascular anatomy. This pilot study evaluates the use of ICG/NIR fluorescence imaging for the assessment of kidney allograft perfusion.

## 2. Materials and Methods

This pilot study is institutional review board approved (CIRB 2017/3027), prospectively recruiting 3 patients undergoing living donor (LDRT, n=1) or deceased donor (DDRT, n=2) kidney transplantations in February 2017. Intraoperative ICG/NIR imaging was used for the assessment of graft perfusion.

All donor kidneys were preserved in histidine-tryptophan-ketoglutarate (HTK) solution and kept in static cold storage, prior to transplantation. The allograft venous and arterial anastomoses were performed, end-to-side to the corresponding recipient external iliac vein and artery. Urinary reconstruction was performed using the extra-vesical Lich-Gregoir ureteroneocystostomy technique over a ureteral stent, which was removed 2 weeks later, and an indwelling bladder catheter was routinely kept for 5 days after surgery. Routine immediate postoperative assessment of kidney allograft perfusion was performed using either a nuclear perfusion or a Doppler ultrasound scan.

Following clamp release after completion of vascular anastomoses, a routine assessment of allograft perfusion was done, which included thorough visual inspection of the kidney and vessels, as well as palpation of the kidney turgidity and arterial pulsations. Indocyanine green was given as an intravenous (IV) bolus at a dose of 0.3mg/kg, and a visual assessment of allograft perfusion using fluorescence imaging was performed after a 1-minute interval. Fluorescence imaging was obtained using the xenon-light based KARL STORZ NIR/ICG System, with VITOM® ICG for high definition recording of the images.

## 3. Results

Kidney transplantations were performed successfully in all 3 cases. The addition of fluorescence imaging did not significantly extend the operative times, nor alter the routine surgical procedures. All cases were single renal artery and single renal vein anastomoses. The LDRT recipient had a preemptive kidney transplant. Both DDRT organs were recovered from the same standard criteria (SCD) deceased donor, who died from a traumatic subdural haemorrhage. The recipient and donor organ characteristics are shown in Table 1.

**Figure 1:**
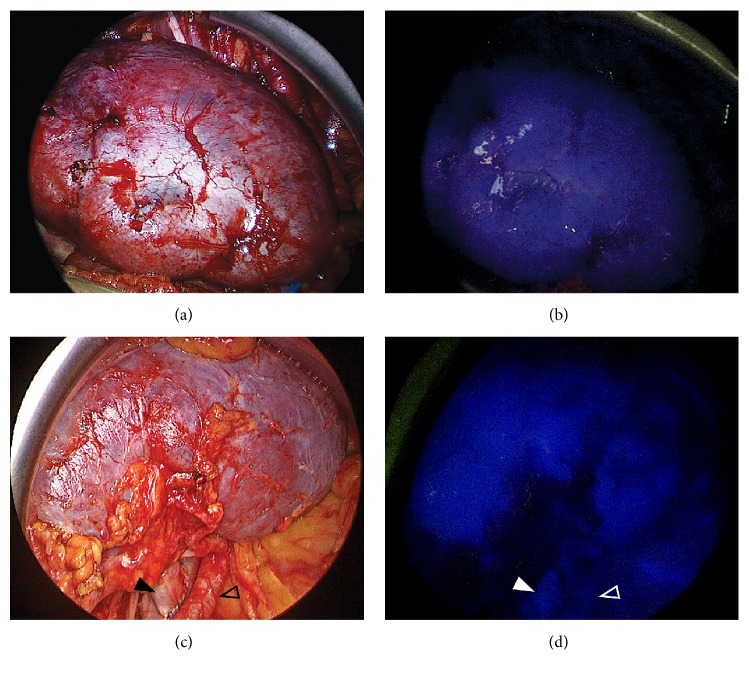
**Intraoperative fluorescence imaging using indocyanine green (ICG)**. Images taken after completion of vascular anastomosis, and after 1-minute following intravenous ICG administration. (a-b) Homogenous global fluorescence observed in graft kidney. (c-d) ICG fluorescence demonstrated in the vessels alongside homogenous graft fluorescence (open arrow represents renal artery; closed arrow represents renal vein).

Homogenous global fluorescence was observed real-time in each graft kidney, and also in the arterial and venous anastomoses, at 1-minute interval following IV ICG administration (see Figure 1). The fluorescence lasted for about 15-20 minutes until the clearance of ICG from the blood. Routine postoperative perfusion scans confirmed good perfusion of the graft kidneys with patent inflow and outflow vessels.

Immediate graft function was observed in 2 cases (1 LDRT and 1 DDRT recipient). One session of haemodialysis was performed for high serum potassium on the first postoperative day of transplant in 1 DDRT recipient, who subsequently had good urine output and serially declining serum creatinine. There were no perioperative or early postoperative surgical complications. In particular, there were no adverse reactions to IV administration of ICG.

## 4. Discussion

The principle of NIR fluorescence imaging revolves around 3 key components, namely, a fluorophore, a light source, and a detector. A fluorophore absorbs energy from the light source and emits fluorescence, which is then picked up by the detector. ICG is cleared by the liver through excretion in bile with a half-life of 3-5 minutes, and the incidence of adverse reactions has been reported at 0.04% [11]. This rapid clearance and good safety profile make ICG the ideal fluorophore for medical imaging. While NIR/ICG imaging has been used in different aspects of surgery, to our knowledge there have only been 3 publications in the English language literature on its use in kidney transplantation.

Sekijima et al. used ICG fluorescence imaging angiography (SPY system; Novadaq Technologies, Inc, Concord, Ontario, Canada) to assess the integrity of vascular anastomoses in kidney and liver transplantations [9]. The authors concluded that fluorescence imaging was useful for the real-time evaluation of vascular reconstructions in solid organ transplantation. In another study, Arichi et al. used a different fluorescence imaging platform (HyperEye Medical System; Mizuho Ikakogyo Co., LTD, Tokyo, Japan) to demonstrate the uptake, steady-state distribution, and clearance of ICG in kidney transplantation, reflecting graft perfusion status [8]. Taking it as a step further, Hoffman et al. used another imaging system (IC-VIEW, Pulsion Medical Systems, Munich, Germany) for the quantitative assessment of transplant kidney perfusion, by measuring the fluorescence intensity as a function of the graft perfusion [10]. The authors also demonstrated patchy fluorescence in kidney allografts, which later had delayed graft function (DGF) postoperatively. It is noteworthy in that study that a large hypoperfusion defect not obvious to the naked eye, caused by a vascular kink, was detected by intraoperative ICG fluorescence imaging, allowing the surgeons to resolve the situation by repositioning the graft in the iliac fossa. In all the studies described, a laser-based light source was used to provide the excitation energy.

In this feasibility study, the KARL STORZ NIR/ICG System used a xenon-light based light source, which did not require the use of laser protective eyewear during surgery. Global fluorescence of the graft kidneys could act as a surrogate for the status of organ perfusion and vascular patency. This was reflected in the postoperative renal perfusion scan and renal function. Nevertheless, these were uncomplicated cases with expected good outcome. The KARL STORZ system provides a qualitative imaging, and quantitative measurement of fluorescence is currently not possible on this platform.

Intraoperative fluorescence imaging may be more useful in complex situations, such as multiple renal arteries, or in cases requiring vascular reconstruction, where identifying perfusion defects or non-fluorescent vessels in real-time may allow timely corrective actions before irreversible damage can occur. The current practice in our institution of performing formal perfusion scan postoperatively may result in a delay in identifying such perfusion defects which are not easily perceptible to the naked eye intraoperatively. Finally, assessing the graft ureteral perfusion may reduce stricture rates of the ureterovesical anastomosis by identifying poorly perfused segment of ureter to be trimmed off.

## 5. Conclusions

In conclusion, NIR/ICG fluorescence imaging is a viable adjunct in renal transplantation for the intraoperative assessment of allograft perfusion and may be useful in complex situations with multiple renal arteries and vascular reconstructions, or when simple visual assessment is deemed inadequate to assess allograft perfusion. Further studies should evaluate its utility in marginal kidneys, and future developments may allow quantitative measurements of fluorescence as a surrogate for Doppler perfusion indices.

## Figures and Tables

**Table 1:** **Recipient demographics and donor organ characteristics**. Donor organs comprise two kidneys from the same deceased donor, and one from a living donor.

**Recipients**	
Male, n	1
Female, n	2
Age (years)	51 (42 – 52)*∗*
Time on dialysis (months)	121 (0 – 200)*∗*
Cause of renal failure	
Glomerulonephritis, n	3

**Donor kidneys**	
Age (years)	47 (47 – 51)*∗*
Cold ischemia time (hours)	6 (1 – 10)*∗*
Warm ischemia time (minutes)	23 (20 – 28)*∗*
Cause of death^*T*^	Traumatic brain injury

*∗* Median (range).

^*T*^ Deceased donor organs.

## Data Availability

The data used to support the findings of this study are included within the article.
